# Distinct Mechanisms of Acetic Acid Inhibition in Thermophilic GH10 and GH11 Xylanases

**DOI:** 10.4014/jmb.2602.02022

**Published:** 2026-04-21

**Authors:** Dan-Gyeong Han, Beom Soo Kim, In Jung Kim

**Affiliations:** 1 Division of Applied Life Sciences, Institute of Agriculture and Life Science, Gyeongsang National University, Jinju 52828, Republic of Korea; 2 Department of Food Science & Technology, Institute of Agriculture and Life Science, Gyeongsang National University, Jinju 52828, Republic of Korea

**Keywords:** Xylanase, Acetic acid, Lignocellulosic pretreatment, Xylan, Inhibitor, Enzyme kinetics

## Abstract

Acetic acid generated during dilute acid or hydrothermal pretreatment of lignocellulosic biomass often acts as a potent inhibitor of glycoside hydrolases (GHs), including xylanases, thereby reducing the efficiency of xylan saccharification. This lowers xylose and xylooligosaccharide yields and overall carbon recovery in lignocellulosic biorefineries. In this study, we compared the inhibition patterns of a GH10 xylanase (TcrGH10) and a GH11 xylanase (TsaGH11) by acetic acid. Both enzymes exhibited only a modest loss of activity (~10–15%) between 0 and 0.8% acetic acid but were almost completely inactivated at 1.0%, suggesting a critical concentration range. Global fitting of kinetic data at 0, 0.3, and 0.75% acetic acid to inhibition models indicated mixed-type inhibition with a predominant non-competitive component for TcrGH10, whereas TsaGH11 predominantly followed an uncompetitive inhibition pattern. These findings elucidate family-specific inhibition behaviors and establish a critical acetic acid threshold, offering guidance for process optimization and the development of acetic acid–tolerant xylanases.

## Introduction

Lignocellulosic biomass is the major structural component of plant cell walls and is composed of cellulose, hemicellulose, and lignin; owing to its abundance of renewable carbon, it is recognized as a highly important feedstock in biorefinery processes [1, 2]. When lignocellulosic biomass is hydrolyzed enzymatically or chemically, various fermentable sugars, such as glucose and xylose, are produced, which can be used as versatile substrates [3]; thus, lignocellulosic biomass is considered a highly promising resource in terms of sustainability and industrial applicability.

Xylan, the major hemicellulosic polysaccharide, is depolymerized by xylanases to yield xylooligosaccharides (XOS) and xylose, which can be utilized as prebiotics [4], fermentation substrates, and platform chemicals[5]. In particular, xylose can be converted into various value-added derivatives, including the sugar alcohol xylitol[6]; therefore, valorizing the xylan fraction is regarded as an important research topic in lignocellulosic biorefineries.

However, in native lignocellulosic biomass, the three-dimensional, highly cross-linked lignin matrix surrounds cellulose and hemicellulose, hindering enzyme access to the polysaccharide substrates and making efficient hydrolysis intrinsically difficult[7, 8]. Therefore, various chemical and physical pretreatment processes are essential to facilitate enzymatic hydrolysis by loosening the biomass structure and improving enzyme accessibility[9]. Although pretreatment generally enhances enzyme accessibility and saccharification yields by disrupting the recalcitrant structure, it simultaneously generates numerous toxic by-products that can inhibit downstream enzymes and microbes[10, 11].

In particular, industrially relevant dilute-acid pretreatment and thermochemical pretreatments (such as steam explosion and liquid hot water treatment) produce substantial amounts of low-molecular-weight toxic compounds, including furan derivatives (furfural, 5-hydroxymethylfurfural), phenolic compounds, and organic acids (e.g., acetic acid and formic acid)[12]. These by-products can perturb the structure of enzymes and the microenvironment around their active sites, thereby decreasing enzymatic activity, lowering saccharification yields, and ultimately increasing enzyme loading and process costs, posing significant constraints on process design[11,13,14].

In xylan, a major hemicellulosic component of lignocellulose, acetyl groups are substituted on side chains and are hydrolyzed during pretreatment to release acetic acid [15]. The released acetic acid remains in the pretreatment liquor and subsequent enzymatic hydrolysis systems[16], and at sufficiently high concentrations it has been reported to inhibit the activities of various glycoside hydrolases (GHs), including xylanases. Thus, acetic acid derived from pretreatment acts as a key inhibitory factor for efficient saccharification of xylan from lignocellulosic biomass, and can limit the yield and productivity of XOS and xylose production processes[17].

Xylanases are key enzymes involved in the deconstruction of lignocellulosic biomass and have been extensively studied in a wide range of microorganisms[18]. Among glycoside hydrolases acting on xylan, the majority of characterized endo-β-1,4-xylanases from bacteria and fungi belong to glycoside hydrolase family 10 (GH10) and family 11 (GH11). GH10 xylanases typically possess a (β/α)_8_ TIM-barrel fold and a relatively broad substrate-binding cleft, whereas GH11 xylanases adopt a β-jelly roll fold with a narrower, more substrate-specific active-site cleft. Because of these structural and mechanistic differences, GH10 and GH11 xylanases often exhibit distinct substrate specificities, catalytic properties, and sensitivities to lignocellulose-derived inhibitors[19, 20].

Previously, we characterized a highly robust thermophilic GH10 xylanase, TcrGH10, and a GH11 xylanase, TsaGH11, as efficient biocatalysts for the hydrolysis of xylan-rich substrates[21, 22]. Both enzymes exhibited high catalytic activity toward beechwood xylan and retained substantial activity at elevated temperatures, making them attractive candidates for application in lignocellulosic biorefinery processes. In the present study, these two structurally distinct xylanases were selected as model enzymes to investigate how acetic acid, a typical lignocellulose-derived inhibitor, differentially affects GH10 and GH11 xylanases at the kinetic and pH-dependent levels.

In this study, we aimed to elucidate the inhibition mechanisms by which acetic acid, often generated during lignocellulosic pretreatment, affects the activities of TsaGH11 and TcrGH10. To this end, we analyzed the reaction rates and kinetic parameters (*e.g.*, *K*_m_ and *V*_max_) of the two xylanases at varying acetic acid concentrations in order to distinguish among competitive, non-competitive, and uncompetitive inhibition. In addition, we evaluated how acetic acid within concentration ranges that can realistically arise under lignocellulosic pretreatment conditions influences the efficiency of xylan hydrolysis. The resulting insights are expected to provide a basis for the family-dependent design of efficient enzymatic saccharification processes for lignocellulose-derived xylan and for establishing process conditions and control strategies that minimize enzyme inhibition by pretreatment-derived by-products.

## Materials and Methods

### Materials

Beechwood xylan used as the substrate was purchased from Biosynth (Switzerland), and the xylanases used in this study were the thermophilic TcrGH10 and TsaGH11 enzymes prepared in our laboratory. Acetic acid and all other reagents used in the experiments were of analytical grade and were obtained from Daejung Chemicals & Metals Co. (Republic of Korea).

### Enzyme Preparation and Protein Determination

Recombinant xylanases TcrGH10 (UniProt ID: A0A3G2WJY4) and TsaGH11 (UniProt ID: I3VTR8) were used for all enzymatic hydrolysis experiments. Both enzymes were heterologously expressed in *Escherichia coli* BL21(DE3) grown in Luria-Bertani (LB) medium supplemented with 50 μg/mL ampicillin and purified by Ni-NTA affinity chromatography followed by size-exclusion chromatography, essentially as described previously for TcrGH10 and TsaGH11[21, 22]. Briefly, protein expression was induced with 0.5 mM isopropyl-β-D-1-thiogalactopyranoside (IPTG) when the cultures reached an OD_600_ of 0.4–0.6, and the cultures were incubated at 18°C overnight. Cells were harvested by centrifugation at 4,000 rpm for 20 min and resuspended in lysis buffer consisting of 50 mM Tris-HCl (pH 8.0), 200 mM NaCl, and 20 mM imidazole. The cell suspension was disrupted by sonication on ice and clarified by centrifugation at 16,000 rpm for 30 min. The supernatant was loaded onto a Ni-NTA affinity column (Qiagen, USA), and His-tagged proteins were eluted with buffer containing 50 mM Tris-HCl (pH 8.0), 200 mM NaCl, and 300 mM imidazole. The eluted proteins were further purified on a Sephacryl S-200 size-exclusion chromatography column (GE Healthcare, USA) equilibrated with 10 mM Tris-HCl (pH 8.0) containing 200 mM NaCl, and the enzyme solutions were concentrated using 30 kDa molecular-weight-cutoff centrifugal ultrafiltration devices (Vivaspin^®^ Turbo 15, Sartorius, Germany). The purified and concentrated enzymes were used for all subsequent assays, and protein concentrations were determined using a bicinchoninic acid (BCA) protein assay kit (Sigma-Aldrich, USA) according to the manufacturer’s instructions.

### Enzymatic Hydrolysis

Enzymatic hydrolysis reactions were carried out with enzyme concentrations of 0.986 μg/mL for TcrGH10 and 1.0 μg/mL for TsaGH11. Unless otherwise specified, reactions were performed in 50 mM sodium acetate buffer (pH 5.0) at 50°C for TcrGH10 and 70°C for TsaGH11, using 0.5% (w/v) beechwood xylan as the substrate. Acetic acid was added stepwise to concentrations of 0, 0.1, 0.3, 0.5, 0.75, and 1.0% (v/v). Each reaction was incubated for 10 min under the conditions specified above, which were based on those previously reported as optimal for the respective enzymes, to evaluate the inhibitory effect of acetic acid on xylanase activity.

The concentration of reducing sugars released during hydrolysis was quantified using the 3,5-dinitrosalicylic acid (DNS) assay. The amount of reducing sugars released was determined by measuring the absorbance at 540 nm and referencing a xylose calibration curve. For inhibition assays, xylanase activity was expressed as relative activity (%) by normalizing the amount of reducing sugars produced in the presence of acetic acid to that obtained under the 0% acetic acid condition, which was defined as 100%.

Enzyme Kinetics and Analysis of Inhibition Mechanism

The inhibition mechanism of acetic acid was elucidated by fitting initial-rate data, expressed as specific activity (U/mg), to the Michaelis-Menten equation (Eq. 1) and to inhibition models based on competitive, non-competitive, mixed, and uncompetitive kinetics (Eqs. 2–5) using nonlinear regression. All equations were included below, and model performance was compared using GraphPad Prism (GraphPad Software, USA).

Michaelis-Menten equation:

$$V=\frac{V_{\max}[S]}{K_{m}+[S]}$$ (1)

Competitive inhibition equation:

$$V=\frac{V_{\max}[S]}{\left(1+\frac{\left[I\right]}{K_{i}})(1+\frac{\left[S\right]}{K_{m}}\right)}$$ (2)

Non-competitive inhibition equation:

$$V=\frac{V_{\max}[S]}{K_{m}(\frac{\left[1\right]}{1+K_{i}})+[S]}$$ (3)

Uncompetitive inhibition equation:

$$V=\frac{V_{\max}[S]}{K_{m}+[S](1+\frac{\left[I\right]}{\alpha K_{i}})}$$ (4)

Mixed inhibition equation:

$$V=\frac{V_{\max}[S]}{K_{m}(1+\frac{\left[I\right]}{K_{i}})+[S](1+\frac{\left[I\right]}{\alpha K_{i}})}$$ (5)

For each condition, including the control without inhibitor (0% acetic acid) and the two selected acetic acid concentrations, the initial-rate data were first fitted to the Michaelis-Menten equation to obtain apparent *V*_max_ (maximum velocity) and *K*_m_ (Michaelis constant) values. Subsequently, the same data sets were analyzed using the enzyme inhibition module of GraphPad Prism to estimate *V*_max_, *K*_m_, and inhibition constants for the different inhibition models. The turnover number (*k*_cat_) was calculated by dividing the *V*_max_ by the molar enzyme concentration, and the catalytic efficiency was determined as the ratio *k*_cat_/*K*_m_.

Model performance was evaluated by comparing the corrected Akaike’s information criterion (AICc) and the residual standard deviation (Sy.x), and the inhibition model with the lowest AICc and smallest Sy.x was considered to best describe the experimental data.

### Structural Analysis and Molecular Docking

To gain structural insight into the inhibition of TcrGH10 and TsaGH11 by acetic acid, molecular docking and visualization analyses were performed. The atomic coordinates for TcrGH10 and TsaGH11 were retrieved from the Protein Data Bank (PDB) codes of 9VK4 and 8IH0, respectively. Before docking, solvent molecules were removed from both structures, and acetate molecules (residue name ACT) were additionally removed from the TsaGH11 structure. Docking of acetic acid to each enzyme was carried out using the CB-Dock2 web server [23]. For each xylanase, the binding cavities predicted by the server were screened for acetic acid-binding poses, and the poses with the most favorable binding energies (Vina scores) were selected for subsequent structural analysis.

The docked complexes were imported into PyMOL (USA) for structural inspection and figure preparation. Protein structures were displayed as molecular surfaces, and the surfaces were colored according to electrostatic potential using the built-in vacuum electrostatics function (red, negatively charged; blue, positively charged; white, neutral). Acetic acid was shown as sticks on the electrostatic surface to visualize its putative binding site on each enzyme.

## Results and Discussion

### Effects of Acetic acid on Xylanase Activity

**Fig. 1:**
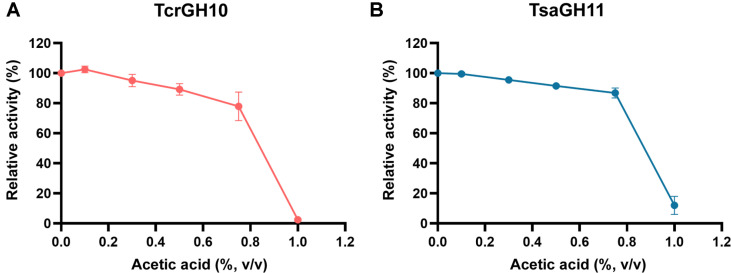
Relative activities of TcrGH10 (A) and TsaGH11 (B) as a function of acetic acid concentration (0–1.0%, v/v). Values represent mean ± SD (n = 3).

To evaluate the effect of acetic acid on xylanase activity, acetic acid was added to achieve the indicated final concentrations. Both xylanases exhibited similar inhibition patterns as the acetic acid concentration increased (Fig. 1). In the range of 0–0.8% (v/v) acetic acid, the relative activities of TcrGH10 and TsaGH11 decreased only gradually, and the loss of activity was limited to approximately 10–15%. However, when the acetic acid concentration reached 1.0% (v/v), the residual activities of both TcrGH10 and TsaGH11 dropped sharply to nearly zero. These observations suggest that the activity loss at acetic acid concentrations below 0.8% (v/v) can be largely attributed to reversible kinetic inhibition, and that the enzymes are not substantially inactivated in this concentration range.

In contrast, at 1.0% (v/v) acetic acid, both enzymes lost almost all activity. To examine whether this abrupt activity loss was associated with pH change, we measured the pH of reaction mixtures containing different concentrations of acetic acid (Table S1). Under these conditions, the reaction mixture at 1.0% (v/v) acetic acid exhibited a pH of 4.16 ± 0.00, which still lies within the pH range where both xylanases are catalytically competent, as shown by our previous study [21, 22]. Thus, the abrupt loss of activity of TcrGH10 and TsaGH11 is not primarily attributable to a pH decrease. Instead, the high concentration of acetic acid may directly affect the enzyme structure. While we cannot fully exclude the possibility of partial irreversible denaturation, the results indicate that the activity loss is more likely associated with direct interactions between acetic acid and the enzyme rather than simple kinetic inhibition due to pH changes.

Although the overall trends were similar, TsaGH11 tended to be slightly more resistant to acetic acid than TcrGH10, as further supported by pH-dependent assays in which acetate was added under conditions favoring either non-ionized acetic acid or dissociated acetate (Fig. S1). At pH 4.0, the non-ionized form (acetic acid) accounted for approximately 85% of total acetic species, whereas at pH 6.0, the dissociated form (acetate) accounted for approximately 95%, allowing us to assess which form more strongly affected each enzyme. For TcrGH10, the activity at pH 4.0 was comparable to or slightly lower than that at pH 6.0, consistent with the greater activity loss of TcrGH10 relative to TsaGH11 observed in Fig. 1.

### Kinetic Analysis of TcrGH10 and TsaGH11 in the Presence of Acetic Acid

Enzyme inhibition is generally classified into competitive, non-competitive, uncompetitive, and mixed inhibition, and these inhibition patterns can be distinguished based on their effects on the kinetic parameters of enzyme reactions. These inhibition mechanisms can be quantitatively characterized by analyzing changes in kinetic parameters, such as *K*_m_ and *V*_max_, within the framework of Michaelis-Menten models. In competitive inhibition, the inhibitor competes with the substrate for binding to the active site of the enzyme, thereby blocking substrate binding and preventing the formation of the productive enzyme-substrate complex. In non-competitive inhibition, the inhibitor binds to an allosteric site distinct from the active site, inducing conformational changes that reduce catalytic activity and lower the maximal reaction rate (*V*_max_) irrespective of whether the substrate is bound in which the inhibitor binds to both the free enzyme and the enzyme–substrate (ES) complex with equal affinity. Uncompetitive inhibition occurs when the inhibitor selectively binds to the ES complex, thereby interfering with the catalytic step; as a result, inhibition is observed only after the substrate has already bound to the enzyme. Finally, mixed inhibition refers to a kinetic mechanism in which an inhibitor binds to both the free enzyme and the ES complex with different affinities.

**Fig. 2:**
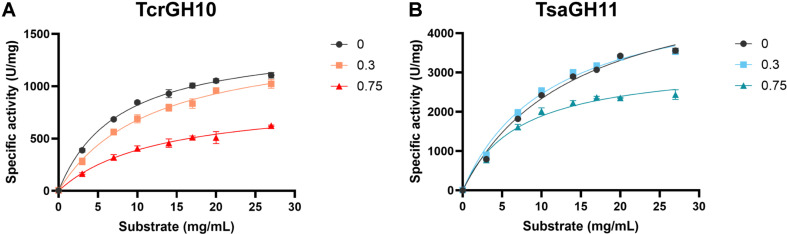
Michaelis-Menten plots for TcrGH10 (A) and TsaGH11 (B) toward beechwood xylan in the presence of 0, 0.3, or 0.75% (v/v) acetic acid. Specific activity (U/mg) was measured at the indicated substrate concentrations (mg/mL). Symbols represent experimental data and solid lines indicate Michaelis-Menten fits. Values represent mean ± SD (n = 3).

To quantitatively characterize the kinetic behavior in the presence of acetic acid in the reversible inhibition regime before complete loss of activity, three acetic acid concentrations were selected: 0% (control), 0.3% (intermediate inhibition), and 0.75% (near-maximal inhibition without full inactivation) (Fig. 2 and Table 1).

In case of TcrGH10, under control conditions without acetic acid, the apparent *V*_max_ and *K*_m_ values for beechwood xylan were 1,445 ± 21.39 U/mg and 7.64 ± 0.34 mg/mL, respectively. At 0.3% (v/v) acetic acid, the apparent *V*_max_ (1,481 ± 59.28 U/mg) remained comparable to the control, whereas the apparent *K*_m_ increased to 11.92 ± 1.48 mg/ml, showing a tendency toward reduced substrate affinity. At the higher inhibitor concentration of 0.75% (v/v), the apparent *V*_max_ decreased markedly to 894 ± 71.72 U/mg (approximately 60% of the control value), while the apparent *K*_m_ slightly increased or remained relatively constant at 12.96 ± 2.37 mg/mL. Overall, these concentration-dependent changes, in which *V*_max_ is preserved and *K*_m_ tends to increase at low inhibitor concentration but *V*_max_ clearly decreases with *K*_m_ remaining relatively stable at higher inhibitor concentration, point to an overall mixed inhibition pattern for TcrGH10. Correspondingly, the turnover number (*k*_cat_) remained stable at 0.3% acetic acid (877.57 ± 5.12 s^-1^) compared to the control (855.84 ± 12.67 s^-1^) but decreased significantly to 529.36 ± 42.59 s^-1^ at 0.75%. Consequently, the catalytic efficiency (*k*_cat_/*K*_m_) declined progressively from 112.0 ± 5.3 ml/mg/s in the control to 73.6 ± 9.6 and 40.8 ± 8.2 mL/mg/s at 0.3% and 0.75% acetic acid, respectively, indicating a substantial loss of enzymatic efficiency due to the inhibitor.

In case of TsaGH11, under control conditions without inhibitor, the apparent *V*_max_ and *K*_m_ values were 5,675 ± 137.01 U/mg and 14.3 ± 0.57 mg/mL, respectively. In the presence of 0.3% (v/v) acetic acid, the apparent *V*_max_ slightly decreased to 5,218 ± 99.81 U/mg, while the apparent *K*_m_ also decreased to 11.26 ± 0.67 mg/mL. At 0.75% (v/v) acetic acid, both kinetic parameters were further reduced, with *V*_max_ dropping to 3,275 ± 207.75 U/mg and *K*_m_ to 7.35 ± 1.30 mg/mL. Thus, the Michaelis-Menten fits showed that increasing acetic acid concentration led to a concomitant decrease in both *V*_max_ and *K*_m_, a pattern that is classically associated with uncompetitive inhibition. The turnover number (*k*_cat_) followed a similar decreasing trend, dropping from 2194.33 ± 52.98 s^-1^ (control) to 2017.76 ± 38.59 s^-1^ and 1266.46 ± 80.33 s^-1^ at 0.3% and 0.75% acetic acid, respectively. Interestingly, due to the simultaneous and substantial reduction in *K*_m_, the calculated catalytic efficiency (*k*_cat_/*K*_m_) appeared slightly higher or comparable in the presence of acetic acid (179.2 ± 11.2 and 172.3 ± 32.4 mL/mg/s) relative to the control (153.4 ± 7.2 mL/mg/s), which is a characteristic kinetic feature often observed in uncompetitive inhibition where the ratio of *k*_cat_ to *K*_m_ is affected by the degree of reduction in both parameters.

Taken together, TcrGH10 exhibits a mixed inhibition pattern in which *K*_m_ tends to increase while *V*_max_ is largely preserved at low acetic acid concentrations but decreases at higher levels, whereas TsaGH11 shows a concomitant decrease in both *V*_max_ and *K*_m_ with increasing acetic acid, consistent with uncompetitive inhibition behavior. Accordingly, the increase in apparent *K*_m_ observed for TcrGH10 at low acetic acid concentrations suggests a competitive-like component in which acetic acid partially interferes with productive substrate binding, whereas the maintenance of *K*_m_ at higher inhibitor concentrations, accompanied by a clear decrease in *V*_max_, indicates an additional non-competitive-like effect on the catalytic turnover step. By contrast, the concomitant decreases in *V*_max_ and *K*_m_ for TsaGH11 can be interpreted as behavior approaching classical uncompetitive inhibition, in which acetic acid may interact preferentially with the ES complex.

### Inhibition Mechanisms of TcrGH10 and TsaGH11 by Acetic Acid

**Fig. 3:**
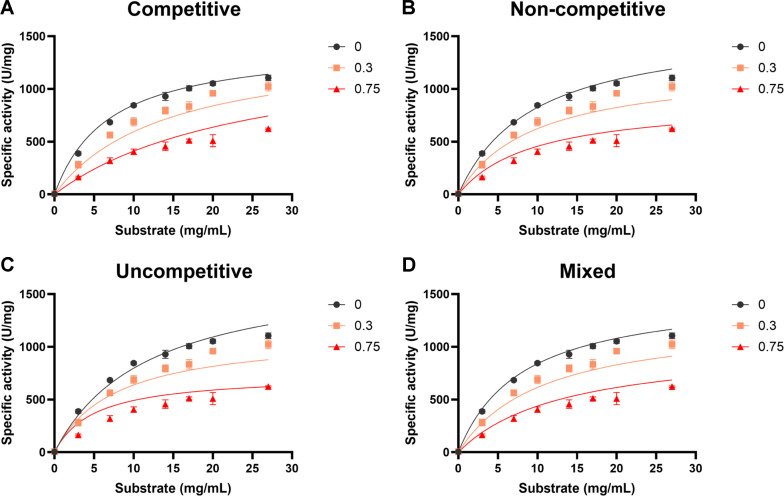
Fits of TcrGH10 inhibition models for beechwood xylan in the presence of acetic acid. Specific activity (U/mg) was measured at the indicated substrate concentrations (mg/ml) with 0, 0.3, and 0.75% (v/v) acetic acid, and the data were fitted to the competitive (**A**) non-competitive (**B**) uncompetitive (**C**) and mixed (**D**) inhibition models, as indicated. Symbols represent experimental data and solid lines indicate the best-fit curves. Values represent mean ± SD (n = 3).

**Fig. 4:**
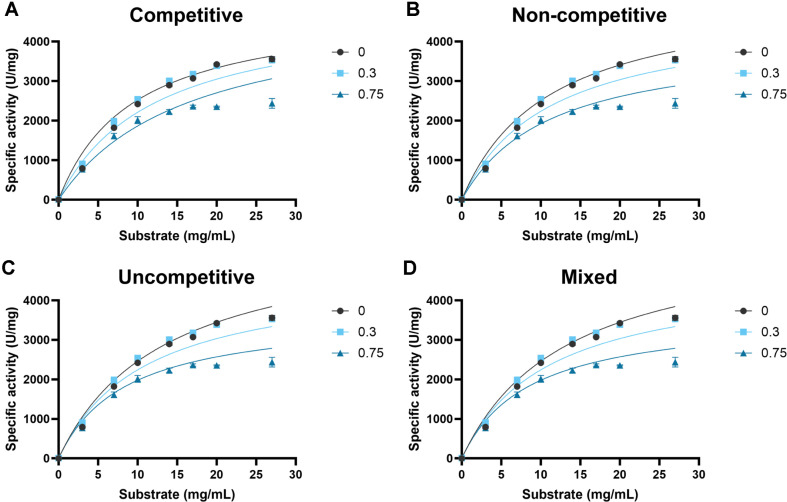
Fits of TsaGH11 inhibition models for beechwood xylan in the presence of acetic acid. Specific activity (U/mg) was measured at the indicated substrate concentrations (mg/ml) with 0, 0.3, and 0.75% (v/v) acetic acid, and the data were fitted to the competitive (**A**) non-competitive (**B**) uncompetitive (**C**) and mixed (**D**) inhibition models, as indicated. Symbols represent experimental data and solid lines indicate the best-fit curves. Values represent mean ± SD (n = 3).

To further elucidate the inhibition mechanism, all the specific activity data obtained at 0, 0.3, and 0.75% (v/v) acetic acid in Fig. 2 were globally fitted to competitive, non-competitive, uncompetitive, and mixed inhibition models derived from Michaelis-Menten kinetics (Figs. 3 and 4), and the quality of fit was compared using R^2^, the residual standard deviation (Sy.x), and the corrected Akaike’s information criterion (AICc) values (Table 2).

In case of TcrGH10, among the tested models, three models including competitive, non-competitive, and mixed inhibition models yielded the lowest comparable AICc values, suggesting a good overall description of the data. In addition, the mixed inhibition model provided the smallest Sy.x and the highest R^2^, indicating that it reproduced the experimental specific-activity profiles most accurately despite the higher model complexity. These combined statistical criteria therefore support a mixed inhibition model with a predominant non-competitive character, rather than a purely competitive or purely uncompetitive mechanism. The kinetic parameters obtained from global fitting to the mixed inhibition model are summarized in Table 3, showing a *K*_i_ of 0.5132 (%, v/v) and an α value of 3.071, with α*K*_i_ noticeably larger than *K*_i_. This trend suggests that acetic acid perturbs the catalytic turnover step more strongly than substrate binding. This parameter pattern supports a mixed-type mechanism in which acetic acid affects both substrate binding (*K*_i_) and catalytic turnover (α*K*_i_), with the non-competitive component being predominant.

In case of TsaGH11, among the tested models, the uncompetitive inhibition model provided the lowest Sy.x and the lowest AICc values, indicating the best overall agreement with the experimental data (Fig. 2). In contrast, the competitive, non-competitive, and mixed inhibition models showed higher Sy.x and AICc values and therefore poorer fits, suggesting that they do not describe the inhibition behavior of TsaGH11 as adequately as the uncompetitive model. In the global uncompetitive inhibition fit for TsaGH11 (Table 3), only α*K*_i_ was defined (1.321), consistent with a canonical uncompetitive pattern in which acetic acid may interact preferentially with the ES complex and reduces both *V*_max_ and *K*_m_.

The relationship between the pH-dependent inhibition results (Fig. S1) and the inhibition mechanisms inferred from the kinetic analyses may be related to structural differences between the two enzymes. GH10 xylanases such as TcrGH10 adopt an open (β/α)_8_ TIM-barrel architecture with a relatively wide substrate-binding cleft, which allows small neutral molecules such as acetic acid to access pockets surrounding the catalytic residues [21]. Because the non-ionized form carries no net charge, it can diffuse more readily into partially hydrophobic regions near the active-site cleft and interact with both the free enzyme and the enzyme–substrate complex. In contrast, GH11 xylanases such as TsaGH11 possess a narrower substrate-binding groove formed by the finger–thumb architecture of the β-jelly-roll fold [22]. This more confined active-site geometry can restrict inhibitor access to the free enzyme, while interactions with the enzyme–substrate complex may become more favorable once substrate binding organizes the local hydrogen-bonding and electrostatic network. These structural differences may therefore explain why TcrGH10 appears more sensitive to the non-ionized form of acetic acid than TsaGH11.

### Molecular Docking

**Fig. 5:**
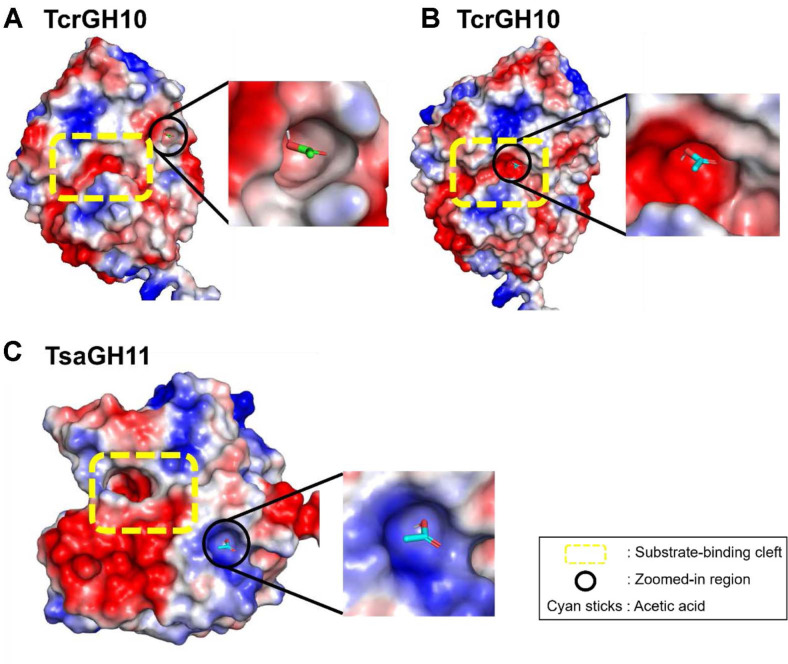
Docking-predicted binding poses of acetic acid on the surfaces of (A and B) TcrGH10 and (C)TsaGH11. Proteins are shown as electrostatic surface representations (red-white-blue), and acetic acid is shown as sticks. The substrate-binding cleft and the predicted binding region are highlighted, with magnified views shown on the right.

To gain a deeper insight into the distinct inhibition mechanisms of acetic acid for the two xylanases, molecular docking was performed. When acetic acid was docked into the TcrGH10 structure using CB-Dock2, the binding pose with the most favorable Vina score was located in a pocket adjacent to the substrate-binding cleft, while the second most favorable pose occupied a position closer to the canonical substrate-binding site (Fig. 5). These docking results suggest that acetic acid can interact primarily with a site neighboring the substrate-binding region, consistent with a non-competitive component, while also potentially associating with the substrate-binding site itself, providing a plausible structural basis for the experimentally observed mixed inhibition pattern with a dominant non-competitive contribution.

In case of TsaGH11, the docking pose with the most favorable binding affinity was located within the canonical substrate-binding cleft. However, this binding mode is more consistent with competitive inhibition, which does not align with the uncompetitive mechanism observed in our kinetic studies. While uncompetitive inhibitors preferentially bind to the enzyme-substrate complex rather than to the free enzyme, our docking was performed with the substrate-free (apo) structure, which poses an inherent limitation in reproducing the actual inhibitory binding mode [24, 25]. Moreover, the highest-scoring pose may not always represent the most relevant binding mode under these conditions. Therefore, this top-ranked pose was excluded from the mechanistic interpretation, and the second-ranked pose was considered a more plausible structural hypothesis consistent with the kinetic data. In this pose, acetic acid was positioned in a pocket adjacent to the substrate-binding site. Because this pocket lies near the active-site cleft, it may be partially formed or become more accessible upon substrate binding, which could allow acetic acid to associate preferentially with the enzyme-substrate complex. This interpretation is consistent with a scenario in which inhibitor binding does not directly block substrate entry but instead affects catalytic turnover after substrate binding. Taken together, these docking results may provide a plausible structural rationale for the distinct inhibition patterns observed in TcrGH10 and TsaGH11. Experimental enzyme-substrate-inhibitor complex structures will be required to confirm the exact inhibition mechanism.

**Fig. 6:**
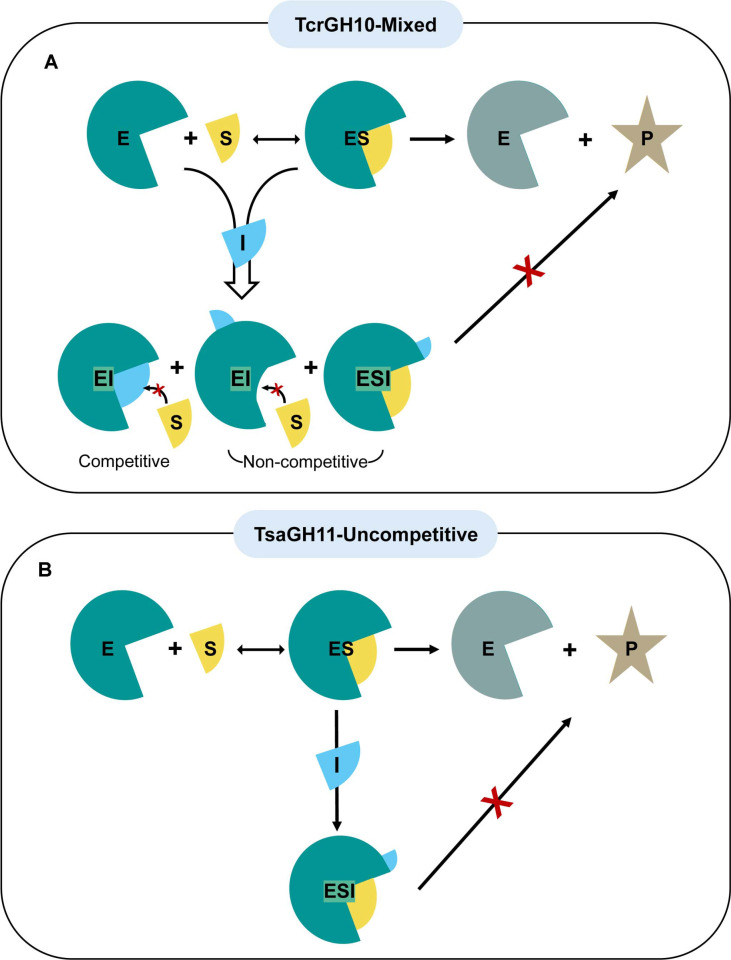
Schematic representation of the inhibition mechanisms of acetic acid for TcrGH10 (mixed inhibition; top) and TsaGH11 (uncompetitive inhibition; bottom). E, S, I, and P denote enzyme, substrate, inhibitor (acetic acid), and product, respectively.

Overall, we propose that, as illustrated in Fig. 6, acetic acid inhibits TcrGH10 through a mixed-type mechanism with a predominant non-competitive component (formation of both EI and ESI complexes), whereas it inhibits TsaGH11 mainly via an uncompetitive mechanism that preferentially targets the enzyme–substrate complex (formation of ESI only).

## Conclusion

In this study, we demonstrate the different inhibition of the two thermophilic GH10 and GH11 xylanases by acetic acid, a major by-product of lignocellulosic pretreatment. Comprehensive analyses revealed that TcrGH10 follows a mixed-type inhibition mechanism with a predominant non-competitive component, whereas TsaGH11 is inhibited mainly through an uncompetitive inhibition pattern. These findings demonstrate that the same pretreatment-derived inhibitor can differentially modulate GH10 and GH11 xylanases, underscoring the importance of controlling acetic acid levels and reaction pH and providing a basis for engineering xylanases with improved inhibitor tolerance.

## Supplemental Materials

Supplementary data for this paper are available on-line only at http://jmb.or.kr.



## Figures and Tables

**Table 1:** Michaelis-Menten parameters of TcrGH10 and TsaGH11 in the presence of acetic acid.

Enzyme	Acetic acid concentration (%, v/v)	*V*_max_ (U/mg)	*K*_m_ (mg/mL)	*k*_cat_ (1/s)	*k*_cat_/*K*_m_ (mL/mg/s)
TcrGH10	0 (control)	1,445 ± 21.39	7.64 ± 0.34	855.84 ± 12.67	112.0 ± 5.3
	0.3	1,481 ± 59.28	11.92 ± 1.48	877.57 ± 35.12	73.6 ± 9.6
	0.75	894 ± 71.72	12.96 ± 2.37	529.36 ± 42.49	40.8 ± 8.2
TsaGH11	0 (control)	5,675 ± 137.01	14.3 ± 0.57	2194.33 ± 52.98	153.4 ± 7.2
	0.3	5,218 ± 99.81	11.26 ± 0.67	2017.76 ± 38.59	179.2 ± 11.2
	0.75	3,275 ± 207.75	7.35 ± 1.30	1266.46 ± 80.33	172.3 ± 32.4

Abbreviations: *V*_max_, maximum velocity; *K*_m_, Michaelis constant; *k*_cat_, turnover number.

Values represent mean ± SD (n = 3).

**Table 2:** Statistical Goodness-of-fit parameters for various inhibition models applied to TcrGH10 and TsaGH11 enzyme kinetics.

Enzyme	Parameter	Competitive	Non-competitive	Uncompetitive	Mixed
TcrGH10	R^2^	0.9659	0.9688	0.9559	0.9709
	Sy.x	66.91	64.06	76.13	63.42
	AICc	208.7	206.6	214.9	208.1
TsaGH11	R^2^	0.955	0.9655	0.9689	0.9689
	Sy.x	254	222.2	211.1	216.3
	AICc	272.7	266.3	263.8	267

Abbreviations: R^2^, coefficient of determination; Sy.x, standard deviation of residuals; AICc, corrected Akaike information criterion.

**Table 3:** Kinetic and inhibition constants for TcrGH10 and TsaGH11 determined from the best-fit inhibition models.

Enzyme	*V*_max_ (U/mg)	*K*_m_ (mg/ml)	*K*_i_ (%, v/v)	α	α*K*_i_
TcrGH10	1515	8.039	0.5132	3.071	1.576
TsaGH11	5752	13.39	-	-	1.321

Abbreviations: *V*_max_, maximum velocity; *K*_m_, Michaelis constant; *K*_i_, inhibition constant for the free enzyme (competitive component); α, factor representing the change in substrate affinity; α*K*_i_, inhibition constant for the enzyme-substrate complex (uncompetitive component). Hyphens (-) indicate parameters not applicable to the specific inhibition model.
