# Supplementary material for: Distinct Mechanisms of Acetic Acid Inhibition in Thermophilic GH10 and GH11 Xylanases
Source: J Microbiol Biotechnol. 2026 Apr 21;36:e2602022. doi: 10.4014/jmb.2602.02022 (PMC13102625; doi:10.4014/jmb.2602.02022)
Supplement: Supplementary file 1 [file jmb-36-e2602022-supple.pdf]

J. Microbiol. Biotechnol. 2026. 36: e2602022

https://doi.org/10.4014/jmb.2602.02022

SUPPLEMENTARY INFORMATION

Distinct mechanisms of acetic acid inhibition in thermophilic GH10 and GH11 xylanases

Dan-Gyeong Han[1], Beom Soo Kim[1], In Jung Kim[2*]

[1]Division of Applied Life Sciences, Institute of Agriculture and Life Science, Gyeongsang National University, Jinju 52828, Republic of Korea

[2]Department of Food Science & Technology, Institute of Agriculture and Life Science, Gyeongsang National University, Jinju 52828, Republic of Korea

[*]Corresponding Author: ij0308@gnu.ac.kr (I.J.K.)

Supplementary Methods

Assessment of pH-dependent inhibition by acetic acid/acetate

Inhibition by acetic acid/acetate was examined in pH-dependent assays using sodium acetate under the enzymatic hydrolysis conditions described in the main text, with the only modification that the reactions were incubated for 1 h. To compare the effects of the non-ionized form of acetic acid and the dissociated acetate ion, the reaction pH was adjusted to either 4.0 or 6.0 and sodium acetate solutions were added such that the final concentrations of the additionally supplied sodium acetate (excluding the 50 mM present in the buffer) were 0, 20, 40, 60, 80, and 100 mM at each pH. For the pH 4.0 series, both the buffer and the sodium acetate solutions used as the inhibitor were adjusted to pH 4.0 before mixing, thereby generating reaction mixtures in which the fraction of non-ionized acetic acid was relatively high. For the pH 6.0 series, the buffer and sodium acetate solutions were adjusted to pH 6.0, creating conditions in which acetate ions were relatively predominant. Statistical analyses were performed using GraphPad Prism (GraphPad Software, San Diego, CA, USA). Data were analyzed by two-way ANOVA (factors: pH and added sodium acetate concentration) followed by Sidak's multiple-comparisons test.

Supplementary Figure and Table

Figure S1

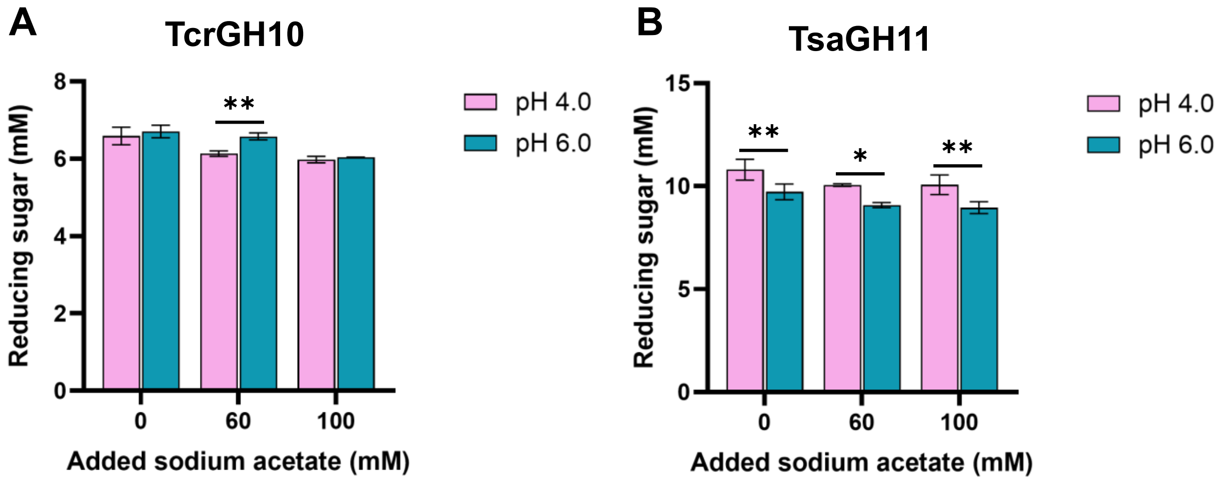

Figure S1. To determine whether xylanase inhibition is caused primarily by non-ionized acetic acid or the dissociated acetate ion, (A) TcrGH10 and (B) TsaGH11 were assayed at pH 4.0 and 6.0, with 0, 60, 100 mM sodium acetate supplemented (in addition to the sodium acetate in the reaction buffer). Data are shown as mean ± SD (n = 3). Statistical significance was determined by two-way ANOVA followed by Sidak's multiple-comparisons test. $*p < 0.05$, $**p < 0.01$.

Table S1. pH values of reaction mixtures containing different concentrations of acetic acid.

Acetic acid concentration (%, v/v)	pH (mean ± SD)
**0**	5.03 ± 0.04
**0.1**	4.83 ± 0.06
**0.3**	4.56 ± 0.02
**0.5**	4.40 ± 0.01
**0.75**	4.27 ± 0.00
**1.0**	4.16 ± 0.00
